# Survey of neurotransmitter receptor gene expression into and out of parental care in the burying beetle *Nicrophorus vespilloides*

**DOI:** 10.1002/ece3.8144

**Published:** 2021-09-23

**Authors:** Christopher B. Cunningham, Daven Khana, Annika Carter, Elizabeth C. McKinney, Allen J. Moore

**Affiliations:** ^1^ Department of Entomology University of Georgia Athens Georgia USA; ^2^ Department of Genetics University of Georgia Athens Georgia USA

**Keywords:** burying beetle, neurotransmitter receptors, *Nicrophorus vespilloides*, reproduction, sociality

## Abstract

Understanding the genetic influences of traits of nonmodel organisms is crucial to understanding how novel traits arise. Do new traits require new genes or are old genes repurposed? How predictable is this process? Here, we examine this question for gene expression influencing parenting behavior in a beetle, *Nicrophorus vespilloides*. Parental care, produced from many individual behaviors, should be influenced by changes of expression of multiple genes, and one suggestion is that the genes can be predicted based on knowledge of behavior expected to be precursors to parental care, such as aggression, resource defense, and mating on a resource. Thus, testing gene expression during parental care allows us to test expectations of this “precursor hypothesis” for multiple genes and traits. We tested for changes of the expression of serotonin, octopamine/tyramine, and dopamine receptors, as well as one glutamate receptor, predicting that these gene families would be differentially expressed during social interactions with offspring and associated resource defense. We found that serotonin receptors were strongly associated with social and aggression behavioral transitions. Octopamine receptors produced a complex picture of gene expression over a reproductive cycle. Dopamine was not associated with the behavioral transitions sampled here, while the glutamate receptor was most consistent with a behavioral change of resource defense/aggression. Our results generate new hypotheses, refine candidate lists for further studies, and inform the genetic mechanisms that are co‐opted during the evolution of parent–offspring interactions, a likely evolutionary path for many lineages that become fully social. The precursor hypothesis, while not perfect, does provide a starting point for identifying candidate genes.

## Graphical abstract

Predicting gene function of nonmodel organisms is necessary to understanding how novel phenotypes arise and how conserved genetic mechanisms are. We tested whether neurotransmitter receptor expression can be predicted from known associations from previously studied organisms during parental care. Some neurotransmitters showed high predictability (serotonin, glutamate), while other did not (octopamine, dopamine).

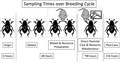

## 1 INTRODUCTION

There is a growing body of research identifying the genetic influences associated with parental care across a wide range of species, particularly gene expression changes (Cunningham, 2020; Royle & Moore, 2019). However, it is difficult to assess the extent that there are common genetic influences across diverse taxa. Parental care is not a simple trait; rather, it is best understood as a behavioral categorization. Parental care can include many distinct, individual behaviors, such as defense of young, construction and maintenance of a nest/reproductive resource, thermoregulation, preparation of food, and direct provisioning of a food resource (Royle et al., 2012). Although collectively these individual behaviors can be considered under the rubric of parental care, the underlying genetic mechanisms may well differ. One proposed solution to developing hypotheses for genes that will underlie components of parental care is the “precursor hypothesis” (Moore & Benowitz, 2019). This hypothesis derives from the suggestion that parental care reflects a repurposing of predictable asocial ancestral traits, such as aggression and resource defense (Tallamy, 1984; Tallamy & Wood, 1986). Breaking parental care into component parts provides hypotheses based on the behavioral traits that are predicted to have been present in ancestral species that lacked care and that were evolutionarily modified. Assuming conservation of mechanism among analogous phenotypes, then the genes underlying parental care behaviors will be those associated with the traits that are co‐opted during the evolution of parental care (Moore & Benowitz, 2019). For example, defense of young is likely to co‐opt genes that influence aggression of species without parental care, while provisioning of young is likely to involve changes in expression of genes that influence foraging or feeding in ancestral species that lacked parental care (Moore & Benowitz, 2019). Therefore, a careful consideration of the individual behaviors that collectively produce parental care can produce a strong expectation of the genes that will underpin those individual behaviors.

Of particular importance to social behavior are neurotransmitters and their receptors as they have a profound and highly conserved influence (Kamhi et al., 2017; Nelson & Trainor, 2007). Thus, neurotransmitters provide an opportunity to test the precursor hypothesis as we can expect associations for nonmodel organisms from known associations in better‐studied taxa. Here, we surveyed the changes of gene expression of multiple neurotransmitter receptors as individuals transition into and out of multiple stages of a complex social behavior, the parental care of the burying beetle *Nicrophorus vespilloides*. Our goal was to test for changes of neurotransmitter receptor gene expression that have known associations with individual behaviors of other species that make up parental care. Addressing this aim allows us to generate new hypotheses about the role of neurotransmitter receptors during parental care. The many changes of individual behaviors that integrate to make up “parental care” suggest that multiple neurotransmitters might be associated with different aspects of parental care to act either independently or synergistically.

The parental care of *N*. *vespilloides* is composed of many different individual behaviors that are encompassed within “parental care,” including behaviors that indirectly and directly benefits offspring (Smiseth et al., 2005; Smiseth & Moore, 2004; Walling et al., 2008). When a small vertebrate carcass is found, a female (or a pair) buries it and performs indirect parental care by preparing the carcass as both a nest and a food resource for developing young. Parents first strip the fur (or feathers or scales) from the carcass, construct a nest within the carcass, and prevent putrefaction of the carcass with antimicrobial excretions. Once carcass preparation has begun, females deposit eggs in the soil around the carcass. When larvae hatch, they crawl into a small cavity in the carcass excised by the parents (Eggert & Müller, 1997; Scott, 1998); the timing of their arrival coincides with the completion of carcass preparation and burial (Oldekop et al., 2007). Indirect parental care continues with carcass maintenance while offspring are present, along with defense of the resource (Walling et al., 2008). Parents also perform direct parental care by feeding dependent, begging offspring predigested carrion. Further direct care for the offspring occurs by excreting enzymes into the larval cavity to preprocess food to make it easily digestible for offspring (Capodeanu‐Nägler et al., 2018). Parental care lasts for 3–4 days, and then, the parent(s) may disperse and the larvae continue to consume the carcass for another few days. Both males and females of this species can provide care by themselves or together with no detectable effect on larval performance (e.g., no difference of larval dispersal mass), and all three parenting environments are observed if pairs are allowed to choose for themselves (Parker et al., 2015). After the carcass is consumed, the larvae also disperse away from the carcass.

**FIGURE 1:**
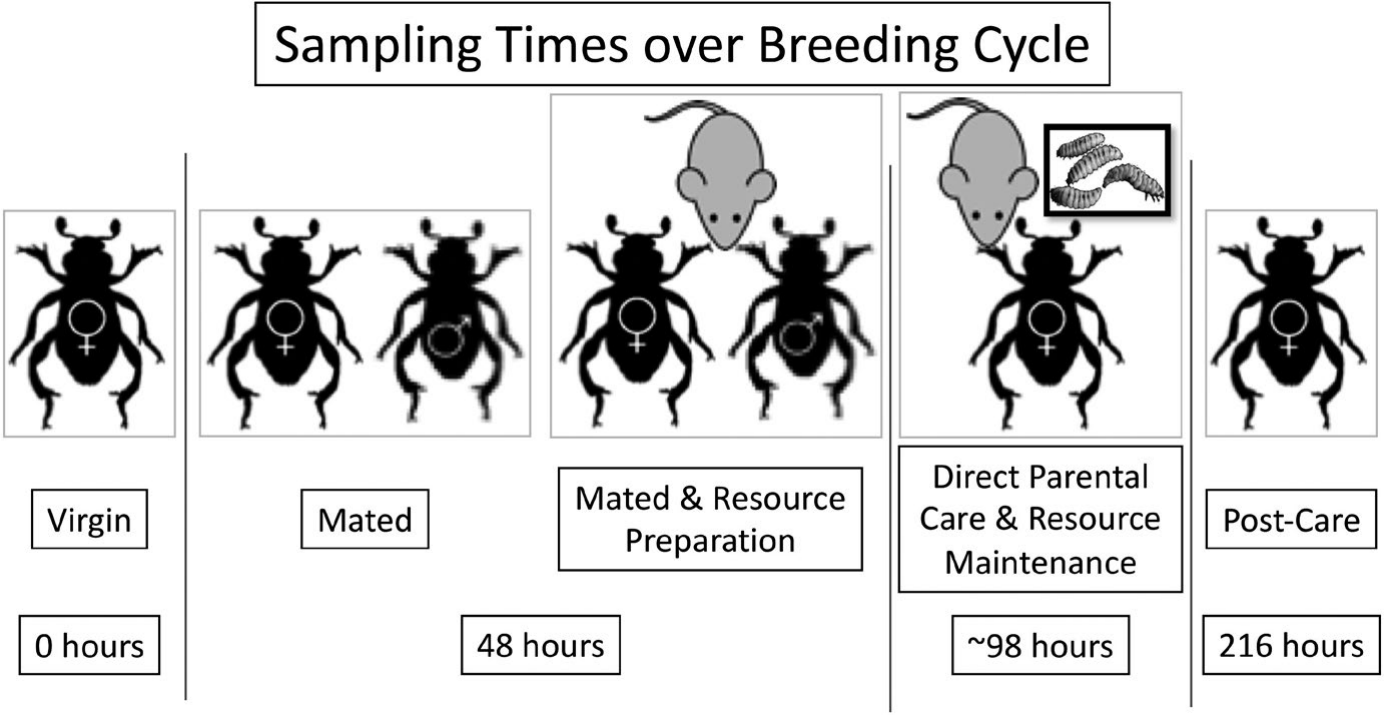
Behavioral categories and times of our samples across a reproductive cycle. Timing starts from placement of males and females together

This stepwise and highly distinct progression through multiple individual behaviors allows us to dissect the associations of individual behavioral transitions with neurotransmitter receptor gene expression (Cunningham et al., 2016, 2017; Parker et al., 2015; Roy‐Zokan et al., 2015). We designed a sampling series that would assess the changes of gene expression as individuals’ transition into and out of parental care (Figure 1). Our treatments span the major behavioral transitions across a reproductive cycle of *N. vespilloides*, from before parental care begins to after parental care ends (Table 1). These transitions were from solitary to mated (Mated) or from solitary to mated with a reproductive resource (Resource Preparation), from resource preparation to active parenting (Direct and Indirect Parental Care), and from active parenting to dispersal after parenting had ceased (Post‐Care). These states therefore represent the gain of experience (social behavior through mating) and the addition of new behaviors (e.g., carcass defense).

**TABLE 1:** Expected changes of individual behaviors over a reproductive cycle as female *Nicrophorus vespilloides* transition into and out of a parental care

Behavioral changes compared with virgin females	Mated	Resource preparation	Direct and indirect parental care^a^	Post‐care	Reference
Sampling within reproductive cycle (hr)	48	48	96	216	
Social tolerance (Mate)	Unchanged	Increased	Increased	Unchanged	Tallamy (2000), Kramer and Meunier (2018)
Defense of resource/offspring	Unchanged	Increased	Highly increased	Unchanged	Tallamy (2000), Trumbo (2007), Kramer and Meunier (2018), Shippi et al. (2018)
Motivation to feed	Unchanged	Decreased	Decreased	Increased	Tallamy (2000), Cunningham et al. (2016)
Sociality (motivation to be around conspecifics)	Unchanged	Decreased	Decreased	Highly decreased	Tallamy (2000), Kramer and Meunier (2018)
Motivation to mate	Unchanged	Equal	Highly decreased	Unchanged	Engel et al. (2014)
Motivation to locomote	Increased	Decreased	Decreased	Slightly increased	Tallamy (2000)
Motivation to care for offspring	Unchanged	Unchanged	Highly increased	Unchanged	Oldekop et al. (2007)

^a^ Larvae appear around 78 hr after pairs were established. Male mates were removed ~60 hr after pairs were established.

Our aim was to select pathways that might be differentially expressed over a reproductive cycle based on the heuristic provided by the precursor hypothesis. This would provide evidence of their possible involvement, but will not provide direct evidence of specific functions. It does, however, test the precursor hypothesis and provide a starting point for further studies of specific gene functions. We surveyed the gene expression of serotonin (5HT), octopamine (OCT), dopamine (Dop), and glutamate (NMDA) receptors during the behavioral transitions across a single reproductive cycle. These neurotransmitters were chosen for their identified functions with other species. For example, the serotoninergic system is associated with periods of increased sociality as serotonin increases (Antsey et al., 2009), as well as the escalation of aggression (Alekseyenko et al., 2010). Serotonin is also needed for parental care of multiple species (Zhao & Li, 2009; Dulac et al., 2014). We assessed all three serotonin receptors of *N. vespilloides* (5HT*r1*, 5HT*r2*, and 5HT*r7*) expecting that they would be differentially expressed during active parenting and resource defense. The octopaminergic system is generally associated with increased aggression (Blenau and Baumann, 2001; Verlinden et al., 2010). We assessed all six receptors of the octopamine/tyramine systems of *N. vespilloides* (*octαr*, *octβr1*, *octβr2*, *octβr3*, *tyrr1*, and *tyrr2*) and again expected these would be differentially expressed when aggression is highest, during resource defense and parenting. The dopaminergic system is also strongly associated with direct parental care (Dulac et al., 2014; Lonstein, 2002; Zhao & Li, 2009) and aggression (Alekseyenko et al., 2013; Rilish & Stevenson, 2014). We assessed all three receptors of the dopamine systems of *N. vespilloides* (*dopr1*, *dopr2*, and *dop2r*) with the same expectation as the octopamine/tyramine receptors. Finally, we profiled the glutamate receptor, *nmdar1*, which is associated with the transition into direct parental care, as seen before with *N. vespilloides* (Parker et al., 2015) and with with other species (Zilkha et al., 2017). One glutamate receptor was assessed (*nmdar1*) with an expectation of changed expression during active parenting.

## 2 MATERIALS AND METHODS

### 2.1 Animal husbandry

We used beetles from an outbred colony of *N. vespilloides* maintained at the University of Georgia originating from and supplemented yearly with beetles from an outbred population at the University of Exeter, Cornwall, UK (Cunningham et al., 2014). We kept beetles in an incubator at a constant 22°C ± 1°C with a 15:9 light:dark cycle. We housed beetles individually as larvae in 9‐cm‐diameter, 4‐cm‐deep circular biodegradable containers with 2.5 cm of moist potting soil (Smart Naturals Happy Frog Potting Mix; Fox Farm, Samoa, CA, USA). We fed all adult beetles two large mealworms (*Tenebrio* sp.) ad libitum once a week after adult eclosion.

### 2.2 Sample collection

We assayed gene expression from female whole head samples collected at specific points across a reproductive cycle of age‐matched individuals (Roy‐Zokan et al., 2015). We have five treatments each with ten biological replicates of single individuals. We started with virgin beetles isolated in individual containers and sampled directly from those containers (treatment 1—Virgins). This behavioral group was nonsocial because the individuals had no contact with other beetles following their own dispersal from the natal nest as larvae. The two reproductive conditions, mated and resource preparation stages, contained females that had been paired with a nonsibling male in a mating box with soil for 48 hr either without (treatment 2—Mated) or with a mouse carcass (treatment 3—Resource Preparation). The mated individuals have the experience of coexisting with another individual and mating but in the absence of the resource required for successful reproduction. The resource preparation condition represents the experience of mating, laying eggs, and coexisting with males while preparing for raising their larvae after being placed with a mouse carcass. Males were removed from the pairing at approximately hour 60 before larvae hatched leaving females to care for offspring under uniparental conditions. Half of families are cared for by uniparental females (Parker et al., 2015). For both the remaining conditions, actively parenting and postcare females were placed in identical mating boxes and set up as the resource preparation individuals. During the active parenting condition, females cared for larvae directly by interacting with offspring and indirectly by spreading oral and anal secretions on the carcass (treatment 4—Direct and Indirect Parental Care). Females were collected for the parental care condition only if they were interacting with the larvae; that is, they were collected only when observed providing direct parental care. Females no longer directly caring for larvae were removed 24 hr before larval dispersal and kept in individual containers for a subsequent 24 hr (treatment 5—Post‐Care). Whole heads were collected and flash‐frozen in liquid nitrogen. Samples were stored at −80°C until RNA extraction using the Qiagen RNeasy Lipid Kit RNA extraction kit followed by cDNA synthesis using the Quantabio each following the manufacturer's instructions (Roy‐Zokan et al., 2015).

### 2.3 Quantitative real‐time PCR (qRT‐PCR)

We identified our candidate genes from *N. vespilloides* using orthologs from *Drosophila melanogaster* and *Tribolium castaneum* and a BLASTp (v2.2.25+; default settings) search (Camascho et al., 2009) of *N. vespilloides* genome (Cunningham et al., 2015). Primer design and validation was conducted as a part of a preliminary study following the protocol outlined in Cunningham et al. (2014). We ensured that each primer generated a single amplicon, had a PCR efficiency of 1.95 or greater, and produced technical triplicates that varied <0.1 cycles using a dilution series of stock virgin cDNA from *N. vespilloides*. Primer sequences for the octopamine/tyramine receptors can be found in Cunningham et al. (2014) and for the serotonin receptors and the glutamate receptor can be found in Benowitz et al. (2017). Primers for the dopamine receptors and their validation results can be found in Appendix S1.

We used an established qRT‐PCR protocol for *N. vespilloides* (Cunningham et al., 2014), using a Roche LightCycler 480 with Roche SYBR I Green Master Mix and 45 cycles of amplification according to the manufacturer's specifications. We ran the biological replicates with technical triplicates and 60°C annealing temperatures. We also performed melting curve analyses at the end of amplification. We used *TATA‐box binding protein* (*tbp*) as the endogenous reference gene. We have previously shown that *tbp* was stable across these behavioral transitions by standardized cDNA input amount into individual reactions (Cunningham et al., 2014; Roy‐Zokan et al., 2015).

### 2.4 Data analysis

We used the ΔΔ*C_T_* method to assess gene expression changes associated with behavioral states by converting raw expression to standardized relative expression values (Livik & Schmittgen, 2001). We performed an overall analysis of variance (ANOVA) to test for any changes of gene expression across the five treatments. Outliers were detected by visual inspection. One Resource Preparation sample for 5HT*r7*, two Post‐Care samples for *dopr1*, and two Post‐Care samples for *dopr2* were removed as outliers. We then tested specific a priori hypotheses using contrast analysis (Rosenthal & Rosnow, 1985), with a one‐degree of freedom contrast comparing Resource Preparation and Parental Care to Virgin, Mated, and Post‐Care treatments. This contrast was chosen as it represents sociality and aggression (resource defense) compared to states without parental care and aggression. We also used Dunnett's test for post hoc pairwise mean differences using the Virgin behavioral state our a priori comparison group. Virgins were chosen as the treatment that represents a basal state that all our treatments begin from when progressing through all necessary behaviors/conditions to successfully complete a reproductive cycle, and so this post hoc tests for changes in any state compared with a baseline. There were ten biological replicates per treatment. All analyses were performed with JMP Pro (v.15.0.0). Data for this paper are available on Dryad (Cunningham et al., 2021).

## 3 RESULTS

### 3.1 Serotonin receptors

**FIGURE 2:**
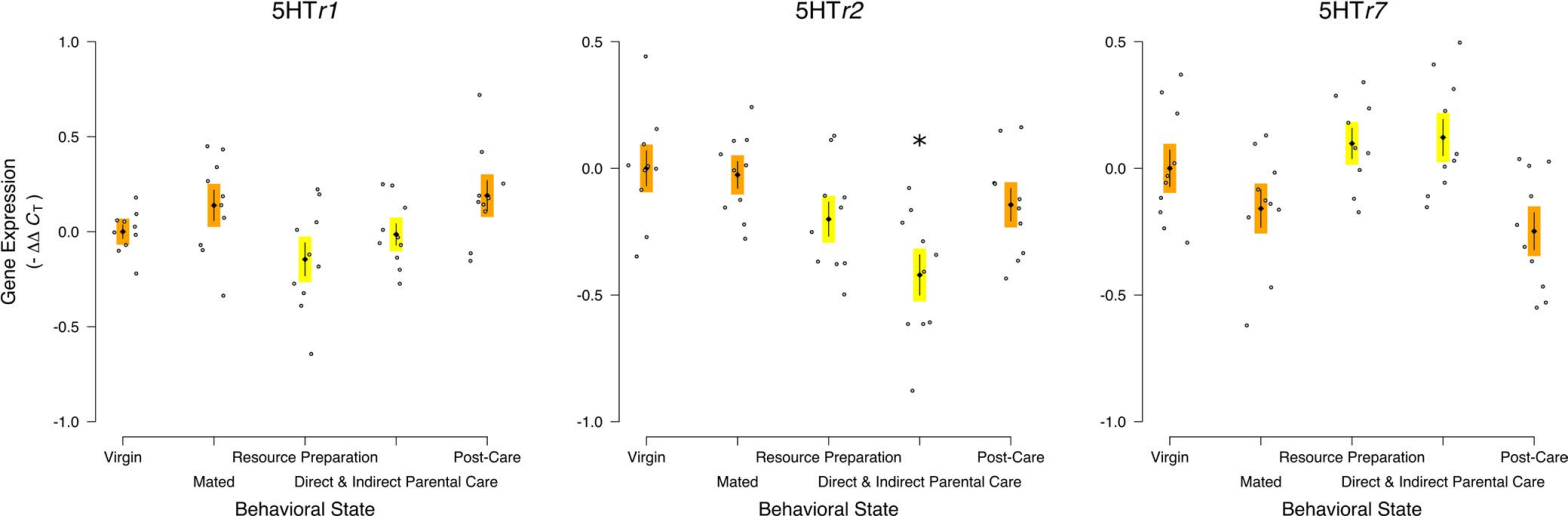
Serotonergic receptor gene expression across the behavioral transitions into and out of parental care of *Nicrophorus vespilloides* supports a strong and conserved role during large changes of sociality/aggression for serotonin receptors. Black diamonds and vertical lines represent means ± *SEM*, while gray dots represent individual sample values. * represents a statistically significant Dunnett's pairwise comparison of means to Virgin. Different colored boxes around the mean and *SEM* indicate a statistically significant a priori contrast between treatments with parenting/aggression and other treatments; otherwise, the same colored boxes indicate the contrast was not statistically significantly different. Sample size—5HT*r1* and 5HT*r2*: 10 Virgins, 10 Mated, 10 Resources Preparation, 10 Direct & Indirect Parental Care, 10 Post‐Care; 5HTr7: 10 Virgins, 10 Mated, 9 Resources Preparation, 10 Direct and Indirect Parental Care, 10 Post‐Care

The expression of *serotonin receptor 1* was statistically significantly different across the behavioral states (5HT*r1*; *F*_4,45_ = 3.635, *p* = .012; Figure 2). The specific contrast showed that there was a statistically significant decrease between the Resource Preparation and Parental Care treatments compared with the other treatments without aggression or parental care (*F*_1,45_ = 5.168, *p* = .028). No treatment was individually statistically significantly different from Virgins.

The expression of *serotonin receptor 2* was statistically significantly different across the behavioral states (5HT*r2*; *F*_4,45_ = 6.332, *p* = .0004). The specific contrast showed that there was a statistically significant decrease between the Resource Preparation and Parental Care treatments compared with the other treatments without aggression or parental care (*F*_1,45_ = 5.531, *p* = .023). Expression of the Direct and Indirect Parental Care state was statistically significantly lower than in Virgins (*D_i_* = 0.181, *p* = .0002).

The expression of *serotonin receptor 7* was statistically significantly different across the behavioral states (5HT*r7*; *F*_4,44_ = 5.263, *p* = .0015). The specific contrast showed that there was a statistically significant increase between the Resource Preparation and Parental Care treatments compared with the other treatments without aggression or parental care (*F*_1,44_ = 99.961, *p* = 7.008 × 10^–13^). No treatment was individually statistically different from Virgins.

### 3.2 Octopamine and octopamine/tyramine receptors

**FIGURE 3:**
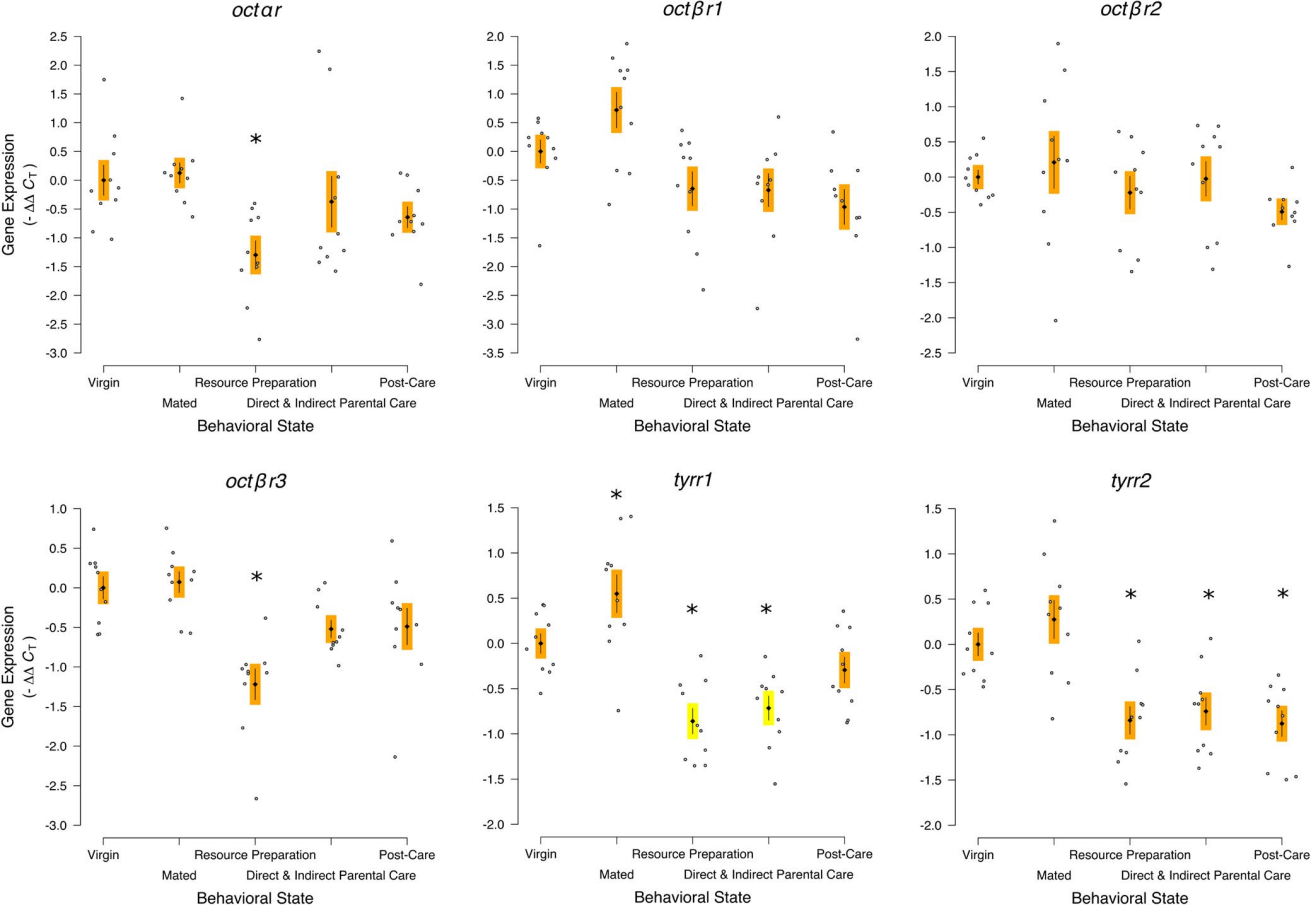
Octopaminergic receptor gene expression across the behavioral transitions into and out of parental care of *Nicrophorus vespilloides* represents a complex picture of receptor expression not strongly associated with any one behavior. Black diamonds and vertical lines represent means ± *SEM*, while gray dots represent individual sample values. * represents a statistically significant Dunnett's pairwise comparison of means to Virgin. Different colored boxes around the mean and *SEM* indicate a statistically significant a priori contrast between treatments with parenting/aggression and other treatments; otherwise, the same colored boxes indicate the contrast was not statistically significantly different. Sample Size: For all genes, 10 Virgins, 10 Mated, 10 Resources Preparation, 10 Direct and Indirect Parental Care, and 10 Post‐Care

The expression of *octopamine alpha receptor* was statistically significantly different across the behavioral states (*octαr*; *F*_4,45_ = 4.228, *p* = .006; Figure 3). The specific contrast showed no statistically significant difference between the Resource Preparation and Parental Care treatments compared with the other treatments without aggression or parental care (*F*_1,45_ = 0.636, *p* = .429). Expression of the Resource Preparation treatment was statistically significantly decreased compared with Virgins (*D_i_* = 0.307, *p* = .007).

The expression of *octopamine beta receptor 1* was statistically significantly different across the behavioral states (*octβr1*; *F*_4,45_ = 5.822, *p* = .0007). The specific contrast showed no statistically significant difference between the Resource Preparation and Parental Care treatments compared with the other treatments without aggression or parental care (*F*_1,45_ = 0.356, *p* = .554). No treatment was individually statistically significantly different from Virgins.

The expression of *octopamine beta receptor 2* was not statistically significantly different across the behavioral states (*octβr2*; *F*_4,45_ = 1.289, *p* = .288). The specific contrast showed no statistically significant difference between the Resource Preparation and Parental Care treatments compared with the other treatments without aggression or parental care (*F*_1,45_ = 3.038, *p* = .088).

The expression of *octopamine beta receptor 3* was statistically significantly different across the behavioral states (*octβr3*; *F*_4,45_ = 9.758, *p* < .0001). The specific contrast showed no statistically significant difference between the Resource Preparation and Parental Care treatments compared with the other treatments without aggression or parental care (*F*_1,45_ = 3.363, *p* = .073). Expression of the Resource Preparation treatment was statistically significantly decreased compared with Virgins (*D_i_* = 0.626, *p* < .0001).

The expression of *octopamine/tyramine receptor 1* was statistically significantly different across the behavioral states (*tyrr1*; *F*_4,45_ = 14.419, *p* < .0001). The specific contrast showed a statistically significant decrease between the Resource Preparation and Parental Care treatments compared with the other treatments without aggression or parental care (*F*_1,45_ = 12.999, *p* = .0008). The Mated treatment was statistically significantly increased compared with Virgins (*D_i_* = 0.014, *p* = .047). The Resource Preparation treatment was statistically significantly decreased compared with Virgins (*D_i_* = 0.179, *p* = .006). The Direct & Indirect Parental Care treatment was statistically significantly decreased compared with Virgins (*D_i_* = 0.324, *p* = .0007).

The expression of *octopamine/tyramine receptor 2* was statistically significantly different across the behavioral states (*tyrr2*; *F*_4,45_ = 11.491, *p* < .0001). The specific contrast showed no statistically significant difference between the Resource Preparation and Parental Care treatment compared with the other treatments without aggression or parental care (*F*_1,45_ = 0.709, *p* = .404). The Resource Preparation treatment was statistically significantly decreased compared with Virgins (*D_i_* = 0.175, *p* = .007). The Direct and Indirect Parental Care treatment was statistically significantly decreased compared with Virgins (*D_i_* = 0.276, *p* = .002). The Post‐Care treatment was statistically significantly decreased compared with Virgins (*D_i_* = 0.311, *p* = .001).

### 3.3 Dopamine receptors

**FIGURE 4:**
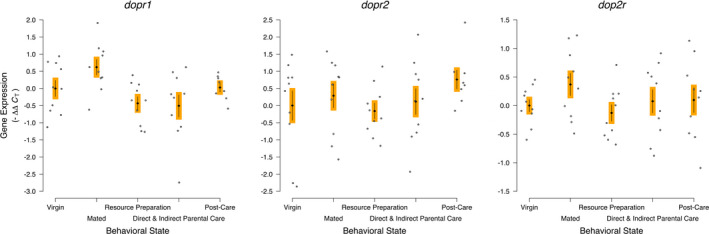
Dopaminergic receptor gene expression was not associated with the behavioral transitions into and out of parental care of *Nicrophorus vespilloides*. Black diamonds and vertical lines represent means ± *SEM*, while gray dots represent individual sample values. Different colored boxes around the mean and *SEM* indicate a statistically significant a priori contrast between treatments with parenting/aggression and other treatments; otherwise, the same colored boxes indicate the contrast was not statistically significantly different. Sample Size—*dopr1* & *dopr2*: 10 Virgins, 10 Mated, 10 Resources Preparation, 10 Direct and Indirect Parental Care, 8 Post‐Care; dop2r: 10 Virgins, 10 Mated, 10 Resources Preparation, 10 Direct and Indirect Parental Care, and 10 Post‐Care

The expression of *dopamine receptor 1* was statistically significantly different across the behavioral states (*dopr1*; *F*_4,43_ = 3.841, *p* = .0094; Figure 4). The specific contrast showed no statistically significant difference between the Resource Preparation and Parental Care treatments compared with the other treatments without aggression or parental care (*F*_1,43_ = 2.835, *p* = .099). No treatment was individually statistically significantly different from Virgins.

The expression of *dopamine receptor 2* was not statistically significantly different across the behavioral states (*dopr2*; *F*_4,43_ = 0.945, *p* = .447). The specific contrast showed no statistically significant difference between the Resource Preparation and Parental Care treatments compared with the other treatments without aggression or parental care (*F*_1,43_ = 0.128, *p* = .722).

The expression of *dopamine DDR2 receptor 2* was not statistically significantly different across the behavioral states (*dop2r*; *F*_4,45_ = 1.071, *p* = .382). The specific contrast showed no statistically significant difference between the Resource Preparation and Parental Care treatments compared with the other treatments without aggression or parental care (*F*_1,45_ = 0.691, *p* = .410).

### 3.4 Glutamate receptor subunit 1

**FIGURE 5:**
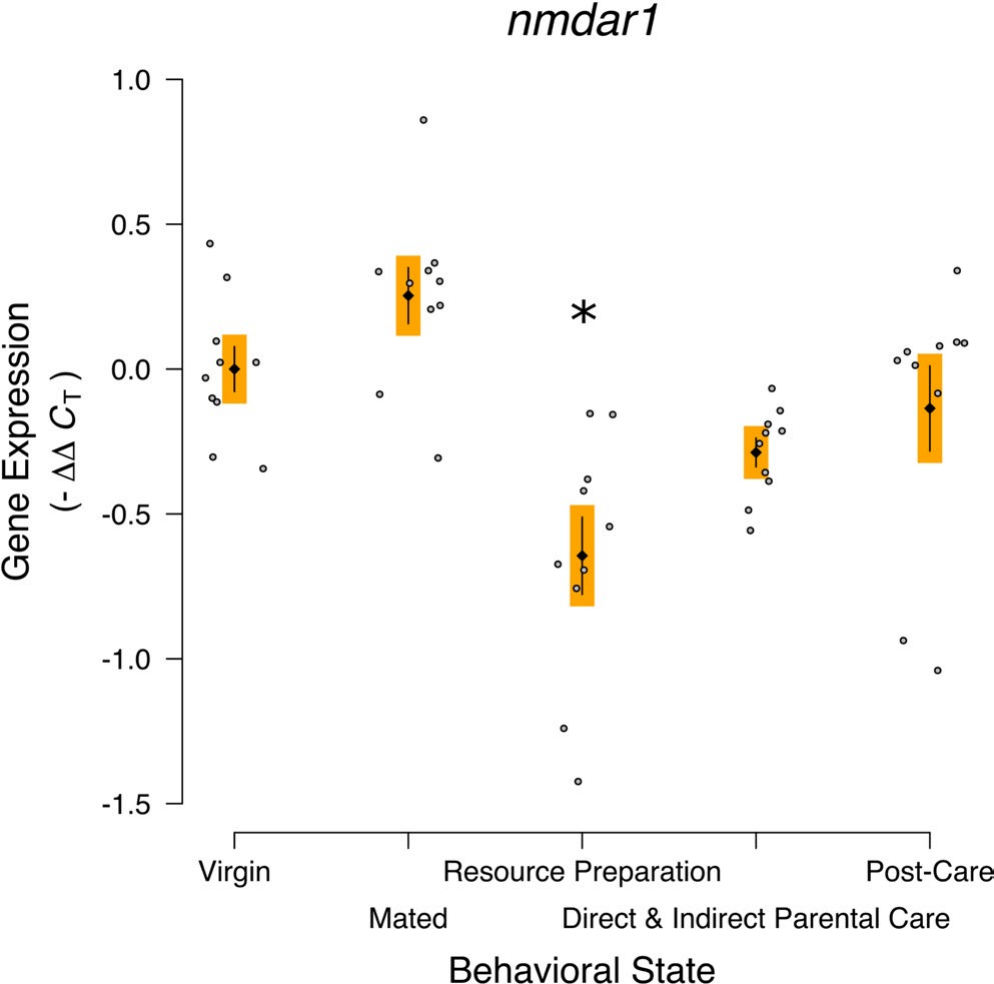
*nmdar1* gene expression across the behavioral transitions into and out of parental care of *Nicrophorus vespilloides* supports an influence on aggression. Black diamonds and vertical lines represent means ± *SEM*, while gray dots represent individual sample values. * represents a statistically significant Dunnett's pairwise comparison of means to Virgin. Different colored boxes around the mean and *SEM* indicate a statistically significant a priori contrast between treatments with parenting/aggression and other treatments; otherwise, the same colored boxes indicate the contrast was not statistically significantly different. Sample Size—10 Virgins, 10 Mated, 10 Resources Preparation, 10 Direct and Indirect Parental Care, and 10 Post‐Care

The expression of *glutamate receptor subunit 1* was statistically significantly different across the behavioral states (*nmdar1*; *F*_4,45_ = 9.893, *p* < .0001; Figure 5). The specific contrast showed no statistically significant difference between the Resource Preparation and Parental Care treatments compared with the other treatments without aggression or parental care (*F*_1,45_ = 1.750, *p* = .193). The Resource Preparation treatment was statistically significantly decreased compared with Virgins (*D_i_* = 0.263, *p* = .0004).

## 4 DISCUSSION

Neurotransmitters and their receptors affect many behaviors across animals (Kamhi et al., 2017; Zilkha et al., 2017). However, broad categories of function of each are generally known that can be used to expect associations with certain behavioral transitions while still remaining in an overall exploratory framework. Here, we assessed the association of serotonergic, octopaminergic, dopaminergic, and glutaminergic receptor gene expression with behavioral transitions into and out of parental care of the burying beetle *N. vespilloides*. All of these neurotransmitter systems are expected to influence social interactions, affiliative and aversive behavior. We expected the strongest pattern changes in expression during Resource Preparation, where resource defense and indirect parental care are highly increased, and Direct & Indirect Parental care, where social interactions are highly increased along with resource defense based on studies from other organisms. Gene expression changes for serotonin, octopamine, and the *nmdar1* receptor(s) had differences across the behavioral transitions into and out of active parenting. In contrast and against expectation, dopamine receptors had no strong differences across the behavioral transition sampled here. All of the serotonin receptors also had differences between the Resource Preparation and Direct and Indirect Parental Care treatments compared with the others. More broadly, the evolution of parent–offspring interactions is hypothesized as a likely evolutionary pathway for animals from solitary to social life history (Kramer & Meunier, 2018). A better mechanistic understanding of parental care will provide a better understanding of one of the likely origins of sociality itself by providing information on the core and lineage‐specific mechanisms possibly used for this transition. Beyond a better mechanistic understanding of parental care itself, this work also supports a conservation of the association between neurotransmitters and complex social behavior in an evolutionarily independent organism.

Serotonin receptor gene expression was associated with the behavioral transitions into and out of parental care, likely due to its association with many of the traits that were expected to change as individuals transition into and out parental care: sociality, mating, feeding, parental care, and aggression (Johnson et al., 2009; Kiser et al., 2012; Voight & Fink, 2015). While all serotonin receptors were associated with a reproductive cycle overall, there was one pairwise comparison with Virgin that was statistically significant. This overall robust association does suggest a meaningful yet complex relationship between serotonin and behavioral changes. The behavioral changes observed as individuals transition into and out of parental care might be heavily influenced by interactions with other neurotransmitters/neuropeptides and not be the result of one neurotransmitter alone (Voight & Fink, 2015). It is also possible that localized associations between behavior and specific areas of the brain influence behavioral changes (Kiser et al., 2012). All three a priori contrast were statistically significant. This result extends the strong support for serotonin's central and conserved role to both social behavior and aggression (Kamhi et al., 2017; Nelson & Chiavegatto, 2001). *Serotonin receptor 2* was associated with Direct and Indirect Care; however, its expression decreased rather than increased. This might be associated with an increase in resource/offspring defense as a decrease in expression of this receptor is associated with increased aggression of insects (Johnson et al., 2009). Behavioral data support this suggestion. Female *N. vespilloides* without mates robustly defend brood against intruding and foreign males that are infanticidal (Shippi et al., 2018). These results extend the association between parental care and serotonin into beetles, reinforcing a conserved and central role of serotonin for sociality and parental care.

Octopamine receptor gene expression was also associated with the behavioral transitions into and out of parental care. Octopamine is positively associated with aggression (Adamo et al., 1995) and reward signaling of insects (Perry & Barron, 2013). The a priori contrast was significant for *tyrr1*. Tyramine is associated with decreased aggression for arthropods (Momohara et al., 2018; Szczuka et al., 2013), so decreasing a receptor could increase aggression. We were able to recapitulate previously observed decrease between *octopamine α receptor* and *octopamine/tyramine receptor 1* expression and a female during Resource Preparation (Cunningham et al., 2014). While *Nicrophorus* females will be aggressive to novel males (Shippi et al., 2018), they still need to be tolerant of a mate. We suggest the *octopamine α receptor* might be playing a role for behavioral flexibility allowing females to discriminate among males as this receptor is associated with memory/learning (Kim et al., 2013; Zhou et al., 2012) and neural activity of underpinning behavioral flexibility (Rein et al., 2013). *octopamine β receptor 3* also has decreased expression in the Resource Preparation stage. *octβr3* is associated with food‐seeking behavior (Zhang et al., 2013) and, here, might decrease the motivation of the female to consume her offspring's food. The *octopamine/tyramine receptor 2* displays a pattern that is most consistent with our expected pattern for sociality. However, there is no documented relationship between *tyrr2* and sociality. Overall, octopaminergic system appears to be very dynamically expressed as individuals transition into and out parental care.

There was no obvious association between the expression of dopamine receptors and parental care despite our expectation from its strong association with vertebrate and insect parental care (Dulac et al., 2014; Panaitof et al., 2016). There was a statistically significant association with *dopamine receptor 1* and the behavioral transitions into and out of parental care, but no a priori contrast was statistically significant and no pairwise comparison with virgins was statistically significant. This might reflect the many individual behaviors that dopamine is associated with that change over a reproductive cycle; mating (Harano et al., 2005), reproduction (Boulay et al., 2001; Sasaki & Harano, 2010), and locomotion (Beggs et al., 2007; Verlinden, 2018). More generally, dopamine is associated with reward signaling within vertebrates and reward signaling plays a large role in vertebrate parental care (Feldman, 2015; Gammie et al., 2016). However, reward signaling within insects is generally assigned to the octopamine system (Perry & Barron, 2013; Verlinden, 2018), which may suggest why we see a lack of a strong association of dopamine with parental care in *N. vespilloides*.

We found that the glutaminergic receptor *nmdar1* was associated with the behavioral transitions into and out of parental care and was reduced during Resource Preparation. The a priori contrast was significant. We suggest this pattern is most consistent with a possible influence on aggression. NMDA receptor inhibitors increase aggression toward intruders of naïve defenders for mammals (McAllister, 1990). Strong associations between glutamate and social interactions have not been identified for insects, but do exist for mammals (e.g., Matveeva et al., 2019). However, we did not see the previously observed association between *nmdar1*, a glutamate receptor, and Direct and Indirect Parental Care (Parker et al., 2015; Zhao & Gammie, 2014). This may reflect sampling or differences in the comparison. Parker et al. (2015) compared mated without a resource and parenting individuals, but the parenting individuals were collected 96 hr after pairing regardless of their behavior at the time. Glutamate is associated both with affiliative social behavior (e.g., Mielnik et al., 2014) and with aggression (Takahashi & Miczek, 2014; Zwarts et al., 2011) and so it may be that individuals were more defensive than parenting in the Parker et al. (2015) study. In support of this, we did see *nmdar1* expression reduced during Resource Preparation. This period when resource defense is strongest perhaps aligns with an increase in territorial aggression seen in rodents (Takahashi & Miczek, 2014). At this stage, the females were with mates and *Nicrophorus* females give a robust defense of the resource (Trumbo, 2007). The reduction in sociality‐associated glutamate would likely manifest through reduced expression within the resource preparation, within active parenting, and especially within the postcare stage when dispersal occurs (Mielnik et al., 2014).

Our main goal was to test for the generality of the precursor hypothesis with respect to neurotransmitters. Our results show that, in general, this is a useful heuristic for identifying genetic targets that might influence parental care and other behaviors. Not all of the genes within a family of neurotransmitter receptors changed. This is to be expected as there are multiple receptors in each family and thus the precursor hypothesis provides a starting point for narrowing functional aspects of gene expression differences associated with behavior. Such narrowing of targets then opens up the possibility of genetic or pharmacological manipulation to move beyond correlation to causation.

## CONFLICT OF INTEREST

We declare no conflicts of interest.

## AUTHOR CONTRIBUTIONS

**Christopher B. Cunningham:** Conceptualization (equal); Data curation (equal); Formal analysis (equal); Investigation (equal); Methodology (equal); Project administration (equal); Visualization (equal); Writing‐original draft (equal); Writing‐review & editing (equal). **Daven Khana:** Investigation (equal); Methodology (equal); Writing‐review & editing (equal). **Annika Carter:** Investigation (equal); Methodology (equal); Writing‐review & editing (equal). **Elizabeth C. McKinney:** Data curation (equal); Investigation (equal); Methodology (equal); Writing‐review & editing (equal). **Allen J. Moore:** Conceptualization (equal); Formal analysis (equal); Funding acquisition (equal); Project administration (equal); Visualization (equal); Writing‐review & editing (equal).

## Supporting information

Appendix S1

## Data Availability

Gene expression data are available on Dryad (Cunningham et al., 2021).
