# Supplementary material for: Survey of neurotransmitter receptor gene expression into and out of parental care in the burying beetle Nicrophorus vespilloides
Source: Ecol Evol. 2021 Sep 23;11(20):14282–92. doi: 10.1002/ece3.8144 (PMC8525115; doi:10.1002/ece3.8144)
Supplement: Supplementary file 1 — Appendix S1 [file ECE3-11-14282-s001.docx]

Appendix S1. Primer Information not reported elsewhere.

Primer Sequences.

*dopr1* -

forward: CTCGACTGCCAGCATCCTCA

reverse: CAGCTACTCTCCTCGTAACCCAT

*dopr2* –

forward: GAGCACACGTGGTTCTTCG

reverse: AGATCACGCACAGATTCAGGAT

*dop2r* –

forward: GGTGAACAAAGCGAGCAAGA

reverse: CACCCAGCAAATCAGGAAAA
